# Generation of Femtosecond Laser-Cut Decellularized Corneal Lenticule Using Hypotonic Trypsin-EDTA Solution for Corneal Tissue Engineering

**DOI:** 10.1155/2018/2590536

**Published:** 2018-04-01

**Authors:** Man-Il Huh, Kyoung-Pil Lee, Jeongho Kim, Soojin Yi, Byeong-Ung Park, Hong Kyun Kim

**Affiliations:** ^1^Bio-Medical Institute, Kyungpook National University Hospital, Daegu, Republic of Korea; ^2^Department of Ophthalmology, School of Medicine, Kyungpook National University, Daegu, Republic of Korea

## Abstract

**Purpose:**

To establish an optimized and standardized protocol for the development of optimal scaffold for bioengineering corneal substitutes, we used femtosecond laser to process human corneal tissue into stromal lenticules and studied to find the most efficient decellularization method among various reagents with different tonicities.

**Methods:**

The decellularization efficacy of several agents (0.1%, 0.25%, and 0.5% of Triton X-100, SDS, and trypsin-EDTA (TE), resp.) with different tonicities was evaluated. Of all protocols, the decellularization methods, which efficiently removed nuclear materials examined as detected by immunofluorescent staining, were quantitatively tested for sample DNA and glycosaminoglycan (GAG) contents, recellularization efficacy, and biocompatibilities.

**Results:**

0.5% SDS in hypertonic and isotonic buffer, 0.25% TE in hypotonic buffer, and 0.5% TE in all tonicities completely decellularized the corneal lenticules. Of the protocols, decellularization with hypotonic 0.25 and 0.5% TE showed the lowest DNA contents, while the GAG content was the highest. Furthermore, the recellularization efficacy of the hypotonic TE method was better than that of the SDS-based method. Hypotonic TE-treated decellularized corneal lenticules (DCLs) were sufficiently transparent and biocompatible.

**Conclusion:**

We generated an ideal protocol for DCLs using a novel method. Furthermore, it is possible to create a scaffold using a bioengineered corneal substitute.

## 1. Introduction

The cornea is the front and outermost part of the eyeball. It serves not only as a mechanical barrier but also as a visual gateway because of its transparency. Transparency is a unique characteristic of the corneal tissue and enables proper visual function [1], but the cornea can be damaged by numerous diseases and injuries that affect its structure. Some causes are irreversible and can be treated by corneal transplantation using human donor tissue [2]. Although corneal transplantation has been successfully performed since the first human corneal transplant in 1905, there are three major disadvantages to this procedure: immunologic graft rejection, possible graft failure, and the lack of donors. Thus, from a clinical perspective, it would be very useful to generate a corneal substitute for the human cornea [3–5].

Currently, tissue engineering methods have been developed as a promising solution for tissue replacement and regeneration. Tissue-engineered corneal equivalents are based on the principle of tissue engineering, which is seeding and proliferating cells within a scaffold. Several scaffolds for corneal equivalents using biological materials have been developed using collagen [6–8] or in combination with glycosaminoglycan (GAG) [9, 10] and fibrin agarose gel [11–13]. Although these approaches have shown some success, there are several limitations compared to the use of human tissue. Decellularized extracellular matrix scaffolds derived from tissues and organs have been successfully used in both preclinical animal studies and human clinical applications. Decellularization methods have unique advantages, such as the use of intact extracellular matrix, no immunologic response, and suitable mechanical strength [14]. Especially, GAG can bind with growth factors and morphogens related with developmental and repair processes, such as VEGF, Wnt, TGF-*β*s, and IGFBP [15, 16]. The retention of the GAG after decellularization is important to regenerate organs [17]. As a possible scaffold for corneal remodeling and as an alternative tissue source for corneal replacement, decellularization of corneal tissue has attracted considerable attention [18–21]. Recently, several research groups have successfully prepared acellular corneal stroma using several detergents and enzymes [14, 21–23]. However, there are two main obstacles that must be overcome before application: one is the shortage of corneal supply and the other is the lack of a standardized decellularized protocol.

Despite increased corneal donation in western countries, the corneal supply does not meet the demand in many other countries. To create allograft rejection-free decellularized cornea, a donor cornea is required. Since 2011, small incision lenticule extraction using a femtosecond laser has become clinically available as an alternative to laser in situ keratomileusis [24]. The corneal lenticules extracted during small incision lenticule extraction can be used for preparation of acellular cornea, rather than a donor cornea.

An ideal decellularization protocol should completely remove cellular material and antigen molecules while retaining the structural and functional proteins of the extracellular matrix without disrupting the overall tissue matrix. However, most decellularization protocols are toxic and destructive to the tissue matrix. A more effective protocol for removing cell components is more destructive to the extracellular matrix composition. Complete cell component removal methods would alter the extracellular matrix composition and cause some degree of ultrastructure disruption. However, there is currently no reliable or standardized protocol for the decellularization of human corneal lenticules, which have different physiological properties with full thickness cornea. Herein, a specific optimized decellularization method for corneal lenticule must be determined to minimize these undesirable effects and achieve complete cell removal.

In this study, we decellularized corneal lenticules created by a femtosecond laser using several reagents with different conditions and compared the efficacy to optimize the protocol. After selecting the optimized method, processed corneas were evaluated with respect to their biological, physical, and ultrastructural properties.

## 2. Methods

### 2.1. Human Corneal Lenticule Preparation

Human corneal tissues were obtained from Santa Lucia International Eye Bank (Manila, Philippines). Approval from the Institutional Review Board of the Hospital Ethics Committee was obtained for the study, and the Declaration of Helsinki was followed. Corneal tissue was positioned under the VisuMax femtosecond laser system (Carl Zeiss Meditec AG, Jena, Germany) using an artificial anterior chamber, and samples were regularly cut to 8 mm wide and 100 *μ*m thick.

### 2.2. Decellularization Processes

Fresh lenticules were decellularized using various concentrations of Triton X-100, sodium dodecyl sulfate (SDS), and trypsin-EDTA (TE) dissolved in hypertonic (100 mM), isotonic (50 mM), and hypotonic Tris buffer (10 mM, pH 7.2). Samples were incubated in each solution for 2 days at 37°C with continuous shaking (100 rpm), and 50 U/mL DNase I (Sigma, MO, USA) and 1 U/mL RNase A (Sigma) were added to each Tris buffer for 1 day at 37°C. Decellularized corneal lenticules (DCLs) were washed with phosphate-buffered saline (PBS) and stored in Optisol™ (Chiron Ophthalmics, Irvine, CA, USA) at 4°C until use (Table 1).

### 2.3. Comparison of Decellularization Efficacy

Decellularization efficacy was compared with histological methods. DCLs were acquired using different three reagents and three tonic buffers (Table 2). Tissues were fixed with 4% paraformaldehyde in PBS (pH 7.4) overnight. Samples were dehydrated through a graded ethanol series, cleared with xylene, and mounted in paraffin. Slides were prepared in 4 *μ*m sections, and the sections were dewaxed, rehydrated, and labeled with Alexa Fluor 488 conjugated anti-vimentin (Abcam, Cambridge, UK) and then counterstained with 1 *μ*g/mL 4′,6-diamidino-2-phenylindole (DAPI). Fluorescence images were acquired using an Eclipse 80i (Nikon, Tokyo, Japan). After processing, we compared the degree of staining.

### 2.4. Measurement of DNA and GAG Contents

Of all protocols, the decellularization methods, which efficiently removed nuclear materials examined as detected by immunofluorescent staining, were quantitatively tested for sample DNA and GAG contents. The lenticules were lyophilized in a freeze dryer (FD5512, IlShin, Gyeonggi-do, Korea) and weighed. The DCLs were digested in 0.2 M sodium phosphate buffer (pH 6.4) containing 125 *μ*g/mL papain (Sigma), 10 mM cysteine hydrochloride (Sigma), 0.1 M sodium acetate (Junsei, Tokyo, Japan), and 2 mM EDTA (Sigma) for 3 h at 65°C as described previously [25]. The DNA content was measured by using a DNA quantitation kit (Bio-Rad, Hercules, CA, USA) according to the manufacturer's protocol. Briefly, 5 *μ*L solution of dissolved lenticules (10 mg/mL) was added to 1 mL TEN buffer (100 mM Tris, 2 M NaCl, 10 mM EDTA, pH 7.4) containing Hoechst 33528 (1 *μ*g/mL). Fluorescence intensity was read using a 460 nm emission filter with a microplate reader (FLUOstar OPTIMA, BMG LABTECH, Ortenberg, Germany). The DNA contents were calculated from a standard curve determined by using calf thymus DNA.

Sulfated GAG in the DCL was measured with a Blyscan™ glycosaminoglycan assay kit (Biocolor, County Antrim, UK). GAG quantification was performed according to the manufacturer's protocol. Absorbance was measured using a VersaMax microplate reader (Molecular Devices, Sunnyvale, CA, USA) at 656 nm.

### 2.5. Electron Microscopy (EM)

The extracellular matrix composition was evaluated by electron microscopic examination. For scanning electron microscopy (SEM), fresh and decellularized tissues were fixed with 2% glutaraldehyde in 0.1 M PBS at 37°C for 1 h, washed with PBS twice, and dehydrated through a graded series of ethanol. All specimens were critical point dried in an HCP-2 (Hitachi, Tokyo, Japan) and coated with platinum using an HCP-2 (Hitachi). Images were observed and captured using an S-4300 (Hitachi). For transmission electron microscopy (TEM), specimens were fixed, dehydrated, and embedded in Epon 812 (Sigma-Aldrich). Ultrathin sections (70 nm) were generated by using an EM UC7 (Leica, Wetzlar, Germany) and stained with 2% uranyl acetate and 1% phosphotungstic acid (Sigma-Aldrich), pH 3.2. TEM images were captured at 100 kV on a HT7700 Bio-TEM (Hitachi).

### 2.6. Evaluation of Transmission of DCLs

To evaluate the optical properties of various DCLs, transmissions of the DCLs were measured with a UV-visible spectrophotometer (Molecular Devices). The DCL was laid on the center of a well surface (48-well plate, Corning Inc., Corning, NY, USA), and wrinkles were removed. The values were measured from 300 to 700 nm wavelength at 10 nm intervals. The graphs were made by subtracting the value of empty wells from those of samples.

### 2.7. Culture of Human Limbal Epithelial Cells (HLECs)

HLECs were cultured from remnant human limbal tissues. After removing the iris, endothelium, and extra conjunctiva, the tissues were incubated with dispase II (4 mg/mL) and trypsin-EDTA for 10 min at 37°C sequentially. Next, the tissues were incubated with 5 mg/mL collagenase I (Worthington Biochemical Co., Lakewood, NJ, USA) for 1 h at 37°C followed by mechanically removing superficial epithelium. After incubation with collagenase I, cells were collected and plated on collagen-coated dishes (IWAKI, Tokyo, Japan) at density of 1 × 10^4^ cells/cm^2^. The cells were remained with CnT-PR and used before passage 3. To isolate keratocytes, stromal tissues were further incubated with 5 mg/mL collagenase I (Worthington Biochemical Co.) for 3 h at 37°C. The cells were collected and plated on a tissue culture dish (Corning) with DMEM (low glucose)/MCDB-201 containing 2% fetal bovine serum (Thermo Fisher Scientific, Waltham, MA, USA), 10 ng/mL epidermal growth factor, 10 ng/mL platelet-derived growth factor, 1 kU/mL leukocyte inhibitory factor, 1% insulin-transferrin-selenium, and 1% antibiotics (Sigma-Aldrich).

In the attachment assay for the DCLs, the cells were reseeded on DCLs at a density of 1 × 10^5^ cells/cm^2^. After 60 min, the cells were fixed with 4% paraformaldehyde and stained with DAPI and fluorescein isothiocyanate- (FITC-) conjugated phalloidin. The images were captured using a Nikon eclipse Ti. The area of cells was calculated using ImageJ software (NIH, Bethesda, MD, USA), and the number of attached cells was counted by unit area (250 × 250 *μ*m). To evaluate the viability of cells on DCL, the cells were seeded in quadruplicate onto 48-well plates (Corning) with 1 × 10^5^ cells/cm^2^. After 24 h, cell growth was measured using the cell counting kit-8 (CCK-8) following the manufacturer's instructions (Dojindo Molecular Technologies Inc., Kumamoto, Japan). The supernatants were transferred into 96-well plates (Corning). Cell viability was measured by reading the absorbance at 405 nm on a 96-well plate reader (VersaMax, Molecular Devices).

To reconstruct a stratified epithelium on DCL, a DCL was laid on a 24-well culture insert (0.4 *μ*m pore size, Corning), and HLECs were seeded at a density of 1 × 10^5^ cells/cm^2^. The cells were cultured for 2 days until confluence. The DCLs were then positioned at the air-liquid interface and further cultured for 2 weeks to induce stratification. The bottom well was filled with 450 *μ*L keratocyte-conditioned medium containing aprotinin (163 *μ*g/mL, Sigma-Aldrich), and the medium was refreshed every 2 days.

### 2.8. Biocompatibility of DCLs

To evaluate the biocompatibility of the DCLs, male ICR mice weighing 25–30 g and male New Zealand white rabbits weighing 1–1.5 kg were used. All animals were treated in accordance with the ARVO statement on the use of animals in ophthalmic and vision research. All animals were maintained and treated according to the Kyungpook National University Animal Care guidelines.

This study was conducted to evaluate the immunogenic potential and tissue response by subcutaneous implantation for 12 weeks in 6-week-old male ICR mice. Healthy animals were randomly assigned into TE-treated groups or the untreated human corneal lenticule group consisting of five animals. The animals were subcutaneously implanted under the dorsal skin. The animals were observed daily for 12 weeks; after the experiments, mice were sacrificed and processed for histopathological evaluation.

Adult New Zealand white rabbits (male, 1–1.5 kg, 6 weeks old) (Daehan Biolink, Seoul, Korea) were used. Recipient animals were anesthetized by intramuscular injection of ketamine (80 mg/kg) and xylazine (10 mg/kg) and 0.5% proparacaine ophthalmic solution (Alcon Laboratories Inc., Fort Worth, TX, USA). Only one eye was operated in each animal. The cornea was incised in the corneal stroma to approximately half depth, and a corneal pocket was created with a spatula. Decellularized corneal discs (diameter of 3 mm) were inserted into the corneal pockets and sutured with 10-0 ethilon for the fixation of corneal lenticule. Two months after implantation, the rabbits were sacrificed with an overdose of pentobarbital sodium.

### 2.9. Reverse Transcript PCR

To evaluate marker gene expression of corneal epithelial cells, total RNA from stratified cells cultured on the DCL was extracted using RNAspin Mini according to the manufacturer's protocol (GE Healthcare Bio-Sciences AB, Little Chalfont, UK). Equal amounts (1 *μ*g) of total RNA were reverse transcribed into cDNA by using a high-capacity cDNA reverse transcription kit (Applied Biosystems, Foster City, CA, USA). The transcribed complementary DNAs were used for real-time polymerase chain reaction (RT-PCR). The PCR primers and annealing temperatures for target genes were designed based on published human gene sequences (Table 3). The amplified products were visualized by 1% agarose gel electrophoresis and ethidium bromide staining, and GAPDH was used as an internal loading control. Images were captured on an ImageQuant LAS 4000 (GE Healthcare).

### 2.10. Statistical Analysis

Data are presented as the means ± standard error of the mean. As all data were shown to be not normally distributed, analysis of variance was used to determine significant differences between samples. *p* < 0.05 was considered to indicate statistical significance.

## 3. Results

### 3.1. Evaluation of Lenticules Decellularized with Various Methods

**Figure 1:**
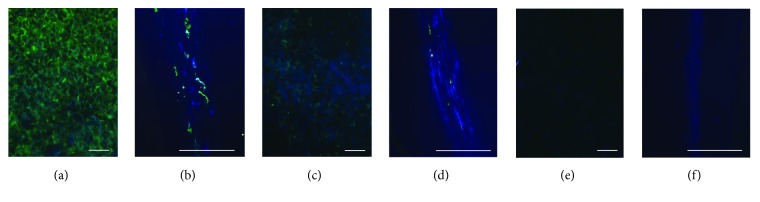
Remained cellular components after decellularization. Remained nuclear and cell component were visualized with DAPI and vimentin after decellularized with hypotonic solution containing 0.5% Triton X-100 (a and b), 0.5% SDS (c and d), and 0.5% TE (e and f). The lenticules were cross sectioned for precise observation, respectively (b, d, and f). DAPI and vimentin stain of TE decellularized lenticules showed no positive signal. Original magnification is ×200 (a, c, and e) and ×400 (b, d, and f). Scale bar indicates 50 *μ*m.

Remaining nucleic acid and cytoskeleton were visualized by immunofluorescence using DAPI and anti-vimentin. 0.5% SDS in hypertonic and isotonic buffer, 0.25% TE in hypotonic buffer, and 0.5% TE in all tonicities showed negative staining of DAPI, while some DCL by 0.5% SDS in hypotonic buffer weakly stained with DAPI (Table 2) (Figure 1).

### 3.2. Comparison with SDS and the TE Decellularizing Method

#### 3.2.1. DNA and GAG Contents

**Figure 2:**
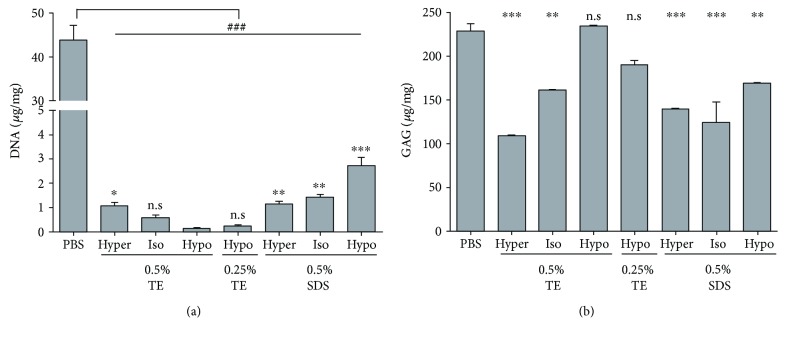
DNA contents and GAG retentions of the DCL. The DNA and GAG contents of DCL under various conditions were measured. The results indicated that the DCL treated with hypotonic 0.5% TE showed the lowest DNA contents (a) and DCL treated with hypertonic 0.5% TE showed the highest GAG contents (b). NC indicates nitrocellulose membrane. Scale bar indicates 50 *μ*m. The values shown are the means ± SEMs (*n* = 4). Statistical significance: ^∗^*p* < 0.05, ^∗∗^*p* < 0.005, and ^∗∗∗^*p* < 0.005 versus hypotonic 0.5% TE. n.s is not significant.

DNA contents were evaluated by Hoechst 33528 staining. DCL in 0.5% hypotonic buffer showed DNA contents of 0.14 ± 0.05 *μ*g per mg of dried DCL, while 0.5% SDS in hypotonic buffer showed as 2.72 ± 0.43 *μ*g. In this study, DCLs of 0.25 and 0.5% TE in hypotonic buffer showed lower DNA contents than the others (Figure 2(a)).

As shown Figure 2(b), remaining GAG contents in 0.5% TE in hypotonic buffer showed the highest level of 234.40 ± 1.10 *μ*g/mg. However, DCLs excluding 0.25 and 0.5% TE in hypotonic buffer showed GAG contents less than 200 *μ*g/mg.

#### 3.2.2. Optical Properties

**Figure 3:**
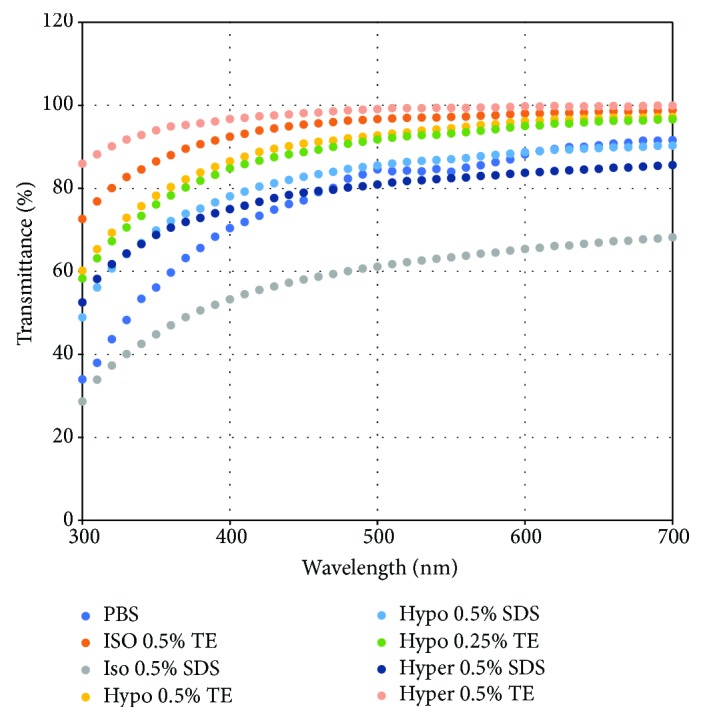
Transmittance of DCLs. Transmittance of the DCL was measured by visible spectrophotometry (wavelength, 300–700 nm). The results indicated that the DCL treated with hypertonic 0.5% TE showed the highest transmittance.

As shown in Figure 3, we examined the transmittance of the DCL. The DCL prepared in hypertonic 0.5% TE showed the best transmittance, while isotonic 0.5% SDS showed the lowest transmittance. Interestingly, DCLs prepared in TE showed better transparency than those in SDS (Figure 3).

#### 3.2.3. In Vitro Biocompatibility

**Figure 4:**
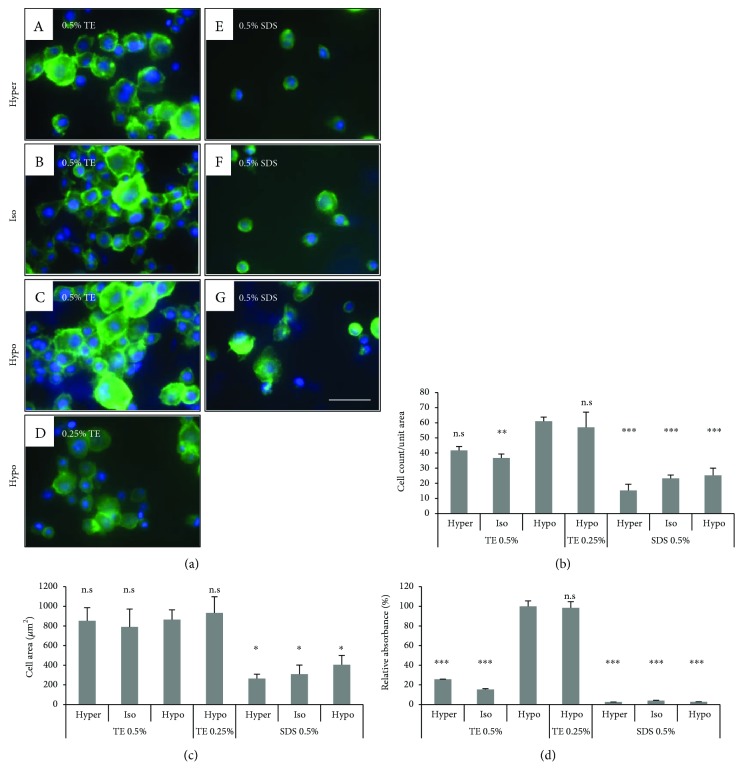
*In vitro* biocompatibility of DCL treated under various conditions. HLEC attachment, spreading, and proliferating activity on the DCLs were evaluated. The cells were seeded on DCL treated with 0.5% TE under various tonic conditions and visualized with DAPI (blue) and phalloidin (green). The count and spreading area of attached cells in TE-treated DCL was higher than on SDS-treated DCL (b and c). Additionally, the proliferating activity of HLECs on DCLs showed the highest rate in hypotonic TE (0.25 and 0.5%). The values shown are the means ± SEMs (*n* = 4). Statistical significance: ^∗^*p* < 0.05, ^∗∗^*p* < 0.005, and ^∗∗∗^*p* < 0.005 versus hypotonic 0.5% TE. n.s is not significant.

**Figure 5:**
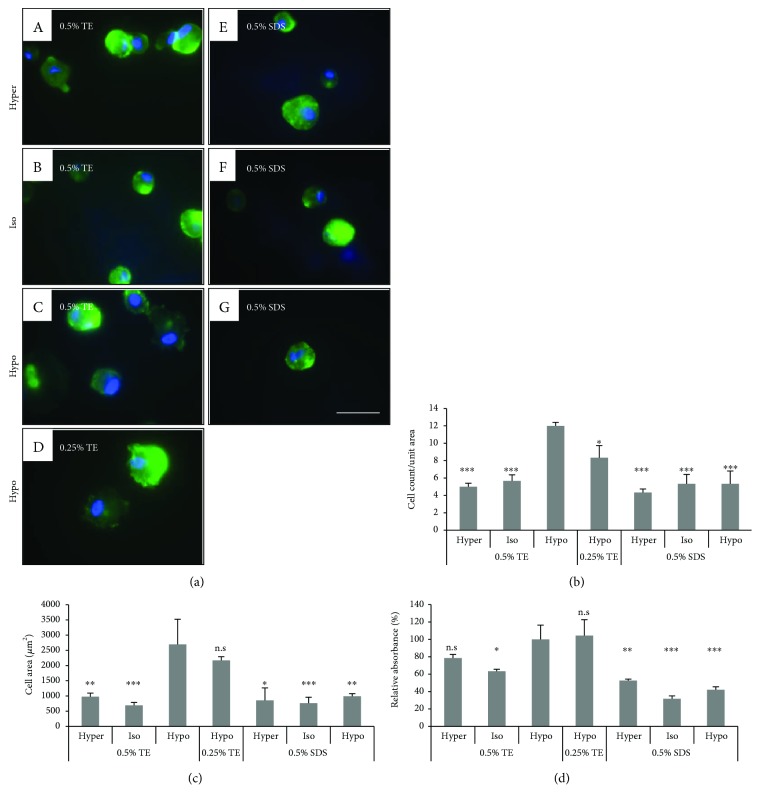
*In vitro* biocompatibility of DCL treated under various conditions. Human keratocyte attachment, spreading, and proliferating activity on the DCLs were evaluated. The cells were seeded on DCL treated with 0.5% TE under various tonic conditions and visualized with DAPI (blue) and phalloidin (green). The count and spreading area of attached cells in TE-treated DCL was higher than on SDS-treated DCL (b and c). Additionally, the proliferating activity of keratocytes on DCLs showed the highest rate in hypotonic TE (0.25 and 0.5%). The values shown are the means ± SEMs (*n* = 4). Statistical significance: ^∗^*p* < 0.05, ^∗∗^*p* < 0.005, and ^∗∗∗^*p* < 0.005 versus hypotonic 0.5% TE. n.s is not significant.

To evaluate the biocompatibility of a HLEC and human keratocyte on the DCLs, we examined cell attachment, spreading area, and proliferating activity. The cells were fixed at 1 h after seeding in DCL at various conditions and then visualized with DAPI and FITC-conjugated phalloidin. TE-treated DCL showed a higher number of attached HLEC than SDS-treated DCL. The numbers of the cells on 0.25% and 0.5% TE in hypotonic buffer were 61 ± 2.75 and 57 ± 10.03 cells/unit area, respectively. Similarly, the spreading area of the cells on TE-treated DCLs was larger than that on SDS-treated DCLs. The cell surface of TE-treated DCL was expanded to 670.56–1097.78 *μ*m^2^/cell, but on SDS-treated DCL was 216.89–500.10 *μ*m^2^/cell (Figure 4). In the case of the keratocyte, TE-treated DCL showed a higher number of attached keratocytes than SDS-treated DCL. The numbers of the cells on 0.5% TE in hypotonic buffer were 12 ± 1.41 cells/unit area. The spreading area of the cells on 0.5% TE-treated DCLs was larger than that on SDS-treated DCLs. The cell surface of 0.5% TE-treated DCL was expanded to 1866.24–4027.43 *μ*m^2^/cell, but on SDS-treated DCL was 462.73–1522.34 *μ*m^2^/cell (Figure 5).

To evaluate proliferation activity, HLEC and keratocyte were analyzed with a CCK-8 assay kit at 24 h after seeding on the DCLs. Notably, proliferating activity of cells on the DCL was only detected in hypotonic TE groups, but showed very low rates in other groups (Figures 4(d) and 5(d)).

### 3.3. Electron Microscopy

**Figure 6:**
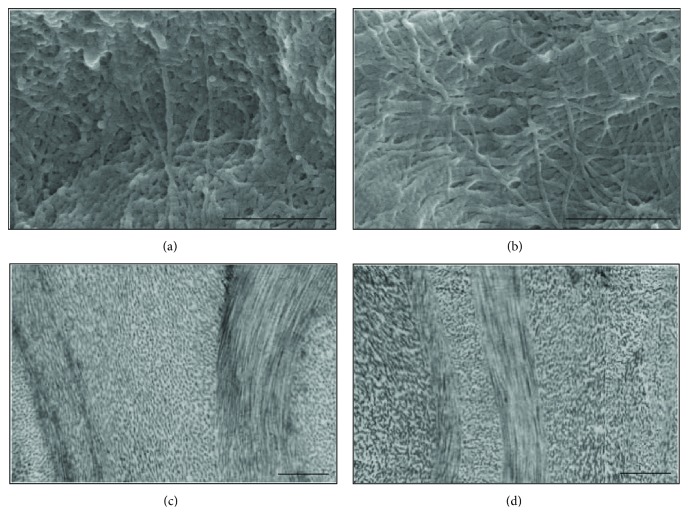
Electron microscopic images of lenticules. Collagen fibril structure of fresh lenticule (a and c) and DCL (b and d). SEM images (a and b) and TEM images (c and d) showing entire collagen microfibrils.

Surface characterization by SEM imaging showed that extracellular matrices in the DCL were not disturbed, and natural collagen bundles were well preserved after decellularization with 0.5% trypsin-EDTA. In the TEM images, collagen fibers are well aligned without damage and showed native matrix orientation (Figure 6).

### 3.4. Examination of Biocompatibility

**Figure 7:**
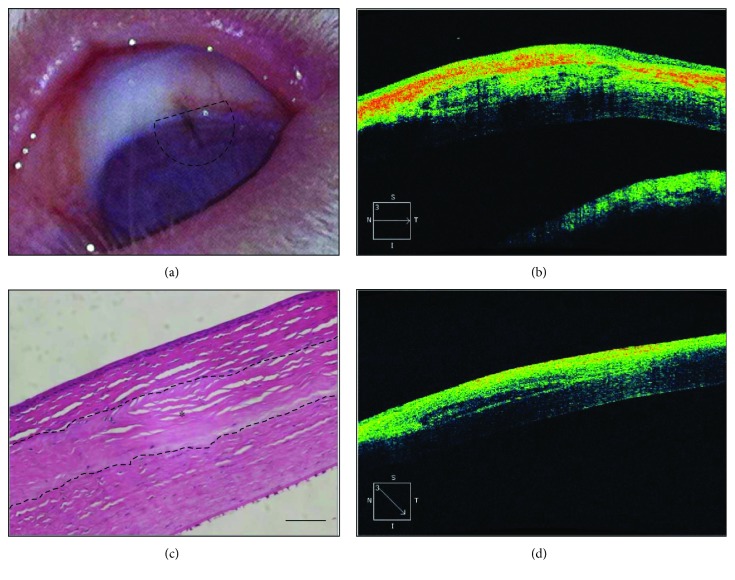
Xenograft of decellularized lenticules into the rabbit corneal stromal layer. DCL was inserted into the stromal layer and fixed with a suture. The slit lamp image shows that the transplanted decellularized lenticule is cleared at 2 weeks after operation. The dotted area indicates the inserted site of the lenticule (a). OCT image showing a transplanted DCL in the stromal layers at 2 (b) and 4 (d) weeks after operation. The DCL was located at the stromal layer. Hematoxylin and eosin staining was performed to detect infiltration of immune cells. There was no infiltration of immune cells in or around the DCL at up to 4 weeks (c). Original magnification is ×200, and scale bar indicates 50 *μ*m (c). ^∗^Implanted DCL.

Biocompatibility testing of a DCL was performed by insertion into the rabbit corneal stromal layer. The DCL was transparent during the implant periods. Optical coherence tomography examination (Spectalis, Heidelberg Engineering, Franklin, MA, USA) was performed at 2 and 4 weeks after surgery on four rabbit eyes and showed that the inserted DCL was settled stably without degradation and haze. Hematoxylin and eosin staining of the cornea collected at 4 weeks revealed no infiltration of inflammatory cells or vascularization, and the DCL remained acellular up to 4 weeks after surgery (Figure 7).

**Figure 8:**
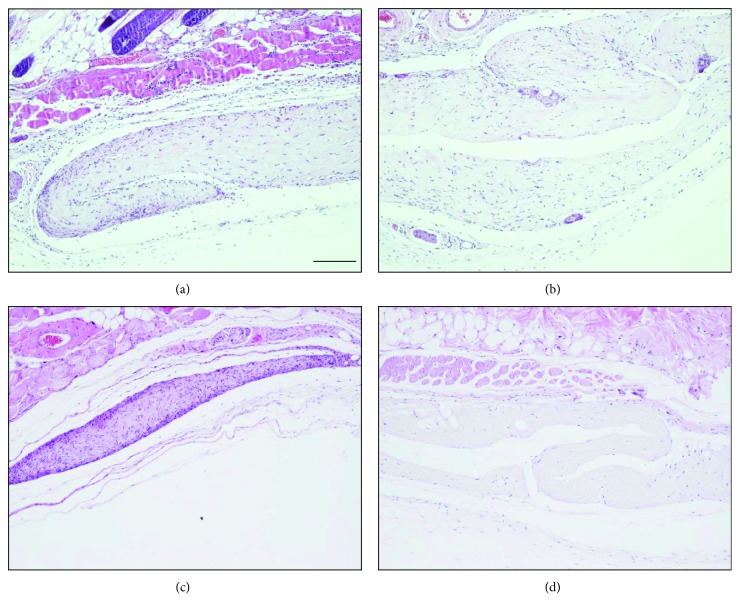
Subcutaneous implantation of the DCL and untreated lenticules in the experimental mouse model. Two weeks after implantation with naive lenticule or DCL, marked inflammatory cells were observed. However, at 12 weeks, DCLs exhibited relatively fewer inflammatory reactions (d) compared to naive lenticules (c). Original magnification is ×100. Scale bar indicates 100 *μ*m.

Unlike interlamellar transplantation, numerous infiltrated inflammatory cells, such as neutrophils and lymphocytes, were detected at 2 weeks after surgery in the subcutaneous implantation model. However, at 12 weeks, the decellularized lenticule exhibited less inflammation with fewer cells than in the untreated model. The untreated lenticule showed numerous infiltrated cells and fibrotic hyperplasia until 12 weeks (Figure 8).

### 3.5. Generation of a Cell Sheet

**Figure 9:**
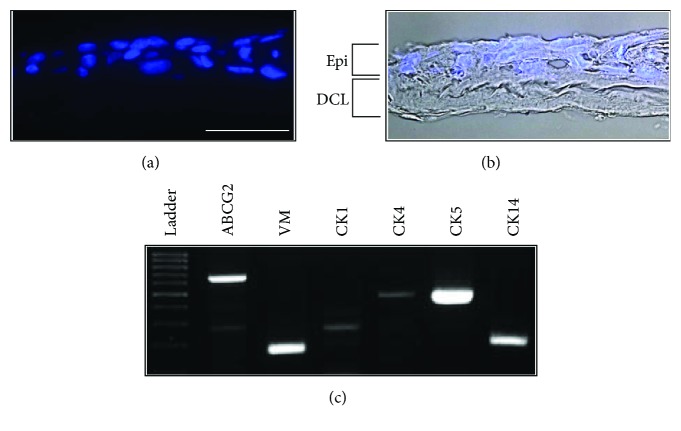
Human limbal epithelial cells on the DCL. Epithelial cells formed a stratified multilayer on the DCL. The cells visualized with DAPI (a) and merged with the bright field image (b). RT-PCR data showed that stratified epithelium cultured on DCL expressed progenitor and corneal epithelial cell markers (c). Scale bar indicates 50 *μ*m.

The ability of the DCL to support corneal epithelial cell growth was examined *in vitro*. After air-liquid interface culture, HLECs formed stratified epithelium containing 3-4 cell layers. We analyzed the gene expression of the constructed cornea anterior lamellar at 2 weeks after air-liquid culture. RT-PCR showed that stratified epithelial cells strongly expressed makers of epithelial cell progenitor cells, such as CK 5, 14, ABCG2, and vimentin (Figure 9).

## 4. Discussion

In this study, we demonstrated the feasibility of femtosecond laser-cut DCL as an alternative scaffold for corneal tissue bioengineering. Femtosecond laser in corneal surgery has several advantages such as its precision and versatility. We obtained very uniform and customized corneal lenticules. Furthermore, the use of femtosecond laser-cut DCL for corneal replacement can overcome the lack of donor cornea because donor cells can be procured during refractive surgery. We should perform various studies to evaluate the safety and efficacy of this method before applying femtosecond laser-cut DCL to clinical cases. We found very promising results. Decellularized corneal tissue as a scaffold for corneal regeneration should maintain the essential properties of the native cornea, such as intact extracellular matrix composition, transparency, and biocompatibility under a harsh decellularization process. However, most decellularization methods use ionic and/or nonionic detergent, which cannot maintain such properties. Many studies have attempted to limit toxicity to maximize the protection of the extracellular composition. However, no comparison studies have optimized the characteristics of the reagents. In this study, we focused on developing an optimized decellularization method for minimizing the toxicity of the reagent without compromising decellularization efficacy. We compared the three reagents most frequently used in each decellularization protocol. A strong ionic detergent such as SDS is commonly used for decellularization of an organ, as it effectively solubilizes cellular membranes and completely removes cells [6, 19, 20, 26, 27]. In the previous report, lenticules which were decellularized with 0.1% SDS combined with 2 U nuclease showed the best decellularizing efficacy [28]. However, SDS causes denaturation of proteins related to the structure of the tissues and causes corneal haze [29, 30]. We also examined that lenticules, which treated with 0.5% SDS, did not maintain morphology of the lenticule in this study. Nonionic detergents, such as Triton X, are also frequently used for corneal decellularization. These detergents are considered milder than ionic treatments, as they target lipid-lipid and lipid-protein interactions, but low decellularization efficacy has been reported [19]. Chelating agents such as EDTA aid in cell dissociation by separating metal ions [29]. These agents are often used in combination with enzymes or detergents because of their lower efficacy for superficial cell removal. Here, we combined trypsin with EDTA, a chelating agent. When we compared the three reagents, TE showed the best efficacy in decellularization. Decellularization efficacy mostly depends upon the tissue, organ, and species of tissue. Unlike a very thick organ or tissues, corneal tissue has a thin, lamellae structure. The decellularization protocol using TE is the most appropriate for preserving the unique collagenous structure.

To increase decellularization efficacy, we evaluated several osmolar conditions with TE. Tonicity can affect the decellularization protocol. Decellularization by immersion in hypotonic or hypertonic solutions can increase decellularization efficacy [31–34]. Hypotonic solutions can lyse cells via osmotic shock and increase reagent penetration, while hypertonic saline can detach DNA from proteins [32]. As shown in our study, TE in hypotonic Tris buffer exhibited the highest decellularization efficacy.

By optimizing the reagents and conditions, we found that hypotonic TE solution showed the best results in the decellularization protocol. To confirm its efficacy and safety, we evaluated hypotonic TE-treated DCL for three properties: extracellular composition, transparency, and biocompatibility. Hypotonic TE-treated DCL was transparent and biocompatible, like native corneal tissue, and preserved the extracellular matrix. Additionally, we successfully constructed an epithelial cell sheet with DCL. Hypotonic TE-treated corneal tissue showed promising results for applications as a scaffold for regenerative cornea.

Taken together, we established a novel method for the decellularization of corneal tissue using a femtosecond laser and suggested that DCL can be used as a scaffold for the partial substitution of diseased cornea including epithelial, stromal, and endothelial conditions.

## Figures and Tables

**Table 1:** Decellularizing process. Corneal lenticules were decellularized by various conditioned solutions. A lenticule incubated with each decellularizing solution, followed by washing with PBS. The lenticules were incubated with DNase I and RNase A to remove nucleic acid. After washing with each tonic buffer, the lenticules were stored in Optisol at 4°C until use.

Number	Process	Time	Temp.
1	Washing with PBS	1 h	37°C
2	Incubation with each decellularizing solution	48 h	37°C
3	Washing with each tonic buffer	1 h	37°C
4	Incubation with DNase I and RNase A	24 h	37°C
5	Washing with each tonic buffer	1 h	37°C
6	Store in Optisol		4°C

**Table 2:** Comparison of decellularizing agents. To extract effective decellularizing conditions, the DCLs were stained with DAPI for detecting remained nucleic acid. “+” showed over 10 positively stained per view, “±” showed few positively stained with DAPI, “−” did not show any nuclear stain in all fields (*n* = 10).

Decellularizing agent	Concentration	Tonicity	DAPI
Triton X-100	0.1%	Hypo	+
		Iso	+
		Hyper	+
	0.25%	Hypo	+
		Iso	+
		Hyper	+
	0.5%	Hypo	+
		Iso	+
		Hyper	+

SDS	0.1%	Hypo	+
		Iso	+
		Hyper	+
	0.25%	Hypo	+
		Iso	+
		Hyper	+
	0.5%	Hypo	±
		Iso	−
		Hyper	−

Trypsin-EDTA	0.1%	Hypo	+
		Iso	+
		Hyper	+
	0.25%	Hypo	−
		Iso	+
		Hyper	+
	0.5%	Hypo	−
		Iso	−
		Hyper	−

**Table 3:** Reverse transcript PCR primers.

Gene	Sequence	Annealing °C	Cycles
GAPDH	5′-GAGTCAACGGATTTGGTCGT-3′	54°C	20
	5′-TTGATTTTGGAGGGATCTCG-3′		
ABCG2	5′-GTTTATCCGTGGTGTGTCTGG-3′	58°C	20
	5′-CTGAGCTATAGAGGCCTGGG-3′		
Vimentin	5′-GAGAACTTTGCCGTTGAAGC-3′	56°C	20
	5′-TCCAGCAGCTTCCTGTAGGT-3′		
CK1	5′-AGGAGGTGGACGTGGTAGTG-3′	54°C	30
	5′-AGGAGGCAAATTGGTTGTTG-3′		
CK3	5′-GGCAGAGATCGAGGGTGTC-3′	59°C	30
	5′-GTCATCCTTCGCCTGCTGTAG-3′		
CK4	5′-CTACAACCTCAGGGGGAACA-3′	55°C	30
	5′-GCTCAAGGTTTTTGCTGGAG-3′		
CK5	5′-CTTGTGGAGTGGGTGGCTAT-3′	56°C	30
	5′-CCACTTGGTGTCCAGAACCT-3′		
CK12	5′-ACATGAAGAAGAACCACGAGGATG-3′	56°C	30
	5′-TCTGCTCAGCGATGGTTTCA-3′		
CK13	5′-GATCCAGGGACTCATCAGCA-3′	56°C	30
	5′-AAGGCCTACGGACATCAGAA-3′		
CK14	5′-TTCTGAACGAGATGCGTGAC-3′	55°C	30
	5′-GCAGCTCAATCTCCAGGTTC-3′		
CK15	5′-GGAGGTGGAAGCCGAAGTAT-3′	58°C	30
	5′-GAGAGGAGACCACCATCGCC-3′		

## Data Availability

The data used to support the findings of this study are available from the corresponding author upon request.
